# Rice bacterial blight resistant cultivar selection based on visible/near-infrared spectrum and deep learning

**DOI:** 10.1186/s13007-022-00882-2

**Published:** 2022-04-15

**Authors:** Jinnuo Zhang, Xuping Feng, Qingguan Wu, Guofeng Yang, Mingzhu Tao, Yong Yang, Yong He

**Affiliations:** 1 College of Biosystems Engineering and Food Science, Key Laboratory of Spectroscopy, Ministry of Agriculture and Rural Affairs, Zhejiang University, Hangzhou, 310058 China; 2 State Key Laboratory for Managing Biotic and Chemical Treats to the Quality and Safety of Agro-Products, Key Laboratory of Biotechnology for Plant Protection, Ministry of Agriculture, and Rural Affairs, Zhejiang Provincial Key Laboratory of Biotechnology for Plant Protection, Institute of Virology and Biotechnology, Zhejiang Academy of Agricultural Science, Hangzhou, 310021 China

**Keywords:** Plant disease, Visible/near-infrared spectroscopy, Attention mechanism, Deep learning, Rice bacterial blight resistance

## Abstract

**Background:**

Rice bacterial blight (BB) has caused serious damage in rice yield and quality leading to huge economic loss and food safety problems. Breeding disease resistant cultivar becomes the eco-friendliest and most effective alternative to regulate its outburst, since the propagation of pathogenic bacteria is restrained. However, the BB resistance cultivar selection suffers tremendous labor cost, low efficiency, and subjective human error. And dynamic rice BB phenotyping study is absent from exploring the pattern of BB growth with different genotypes.

**Results:**

In this paper, with the aim of alleviating the labor burden of plant breeding experts in the resistant cultivar screening processing and exploring the disease resistance phenotyping variation pattern, visible/near-infrared (VIS–NIR) hyperspectral images of rice leaves from three varieties after inoculation were collected and sent into a self-built deep learning model LPnet for disease severity assessment. The growth status of BB lesion at the time scale was fully revealed. On the strength of the attention mechanism inside LPnet, the most informative spectral features related to lesion proportion were further extracted and combined into a novel and refined leaf spectral index. The effectiveness and feasibility of the proposed wavelength combination were verified by identifying the resistant cultivar, assessing the resistant ability, and spectral image visualization.

**Conclusions:**

This study illustrated that informative VIS–NIR spectrums coupled with attention deep learning had great potential to not only directly assess disease severity but also excavate spectral characteristics for rapid screening disease resistant cultivars in high-throughput phenotyping.

**Supplementary Information:**

The online version contains supplementary material available at 10.1186/s13007-022-00882-2.

## Background

Rice bacterial blight (BB), caused by *Xanthomonas campestris pv. Oryzae* (*Xoo*), has been one of the most hazardous and prevalent plant diseases in major rice-producing countries [1, 2]. With the sprawl of bacterial blight in the field, ruinous damage of rice yield and grain quality is inevitable which causes huge economic loss and brings food safety problems [3]. BB pathogens usually choose leaf wounds to invade and the infected rice leaf tends to acquire developmental wilted area on both edge and center consequently resulting in the deficiency of photosynthesis and the decrease of crop production. Although the usage of specialized pesticides would terminate the lethal pathogens, during the practical operation it was unavoidable to pollute the ecological environment. Hence, for effectively managing and minimizing the effect of rice disease without environmental contamination, breeding rice disease resistant cultivar becomes essential. Researchers have spared no effort in mining gene locations/markers related to rice disease resistance ability and exploring the mechanism of genetic resistance [4–7]. Howbeit, cultivating the novel rice disease resistant species requires multi-years planting and screening experiments over plenty of rice genotypes to verify the authentic antipathogenic ability. In conventional practice, rice breeders are supposed to manually measure the lesion length after bacteria inoculation which gives a rise to tremendous labor cost, low efficiency, and severe human error [7, 8]. In addition, from the perspective of plant phenotypic study, proper research about dynamically monitoring BB lesion growth is scarce. In virtue of automatic BB disease severity assessment and sustainable phenotype acquisition, rapid and precise bacterial blight resistant cultivar selection would be achievable as well as uncovering disease resistant regularity, which in return benefit the advance of rice breeding.

Visible and near-infrared hyperspectral image contains both spatial and spectral information with hundreds of narrow and contiguous bands formed a 3D data cube [9]. With the advantage of the non-destructive and informative characteristic of visible/near-infrared spectrum, promising results and methods in plant phenotyping have been made [10–15]. By building discriminant and regression models based on contiguous and narrow hyperspectral data, diverse plant disease is correctly determined and quantified. Furlanetto et al. developed a procedure for detecting rust disease of soybean using spectral analysis, and the validation accuracy of severity classification reached 82.51% [12]. Feng et al. investigated data fusion of multisource spectral data for disease early detection and the result of the final comparison showed that discriminant model based on visible/near-infrared spectrum achieved the best performance [14]. Bendel et al. applied hyperspectral images to detect black sigatoka in banana leaves with a prediction accuracy of 98% while presenting a contributory spectral band range [16]. Apparently, specific responses in spectral reflectance which are related to biotic and abiotic stresses are readily distinct [17]. Visible/near-infrared spectrum provides a powerful tool to assess plant vitality, stress state, and disease category [15]. Nevertheless, when it comes to plant disease phenotyping, more attention is paid on the early detection or classification of disease at single time point rather than dynamic surveillance of the symptom. For disease resistance studies, pathogens are intentionally infected as an experimental stress so as to screening genotypes from their phenotype disparity after inoculation. Variation pattern of lesion regions under time effect is the golden criteria that specifically defines the disease resistant ability. Time-series rice leaf phenotyping is conducible to unveil the growing pattern of BB lesion benefiting the mechanism study of resistant cultivar. And through disease severity ascertainment, both plant breeders and farmers attain credible references to formulate further strategy. Also, redundant features within the hyperspectral bands restrict the rapid and low-cost application from the perspective of practical conditions. Concise spectral combination calculated by retaining some key information of spectrum is going to accelerate the plant disease research progress in a more efficient way.

Deep learning algorithm which is known for its powerful feature extraction and utilization capability is preferred to drastically exploit potential spectral features and reserve pivotal spectral bands. Combined with deep learning algorithm, several studies have already put their focus on disease classification, disease localization with infected leaf spectrum [18–20]. Barbedo et al. augmented their plant disease image database by combining the individual lesions and spots on every image, and their convolutional neural network fulfilled a performance improvement for disease identification [21]. Zhou et al. proposed a progressive detection model for vegetable disease through locating the interested region first, which provided an impressive perspective that it was possible to achieve superior results with the help of innovative model structure [22]. Moreover, Bari et al. put the faster region convolutional neural network (Faster-RCNN) into application to diagnose the rice leaf disease [23]. Different from the whole image classification, the capability which was displaying the disease location further improved the identification accuracy to 99.25%. In general, well-designed deep learning models apparently manifested impressive capability with plant disease.

In this paper, time-series visible/near-infrared spectrum combined with deep learning algorithm was integrated to determine the rice bacteria blight severity and distinguish the BB resistant cultivar. Inspired by the thought in Hu’s study [24], attention mechanism inside the deep learning model was performed to mine the most essential spectral information response to BB infection for assisting high-throughput BB resistant breeding development. The detailed content/objective of this paper were as follows: (1) to build a robust and accurate rice leaf bacterial blight severity estimation model based on time-series VIS–NIR spectrum and deep learning algorithm; (2) to mine intrinsical and refined leaf spectral feature index related to disease severity through the attention mechanism; and (3) to explore the disease severity variation pattern of different rice genotypes and screen BB resistant cultivar with the assist of the leaf spectral index.

## Methods

### Sample preparation

Three rice varieties, namely IR24, 3A26, and 4A37, with diverse BB resistance were chosen to cultivate in this study. First of all, IR24 (*Orazy sativa *ssp.* Indica*) is an elite cultivar designed by International Rice Research Institute but it is highly susceptible to the rice BB. Secondly, the other two varieties which possess BB resistance are constructed by introducing two quantitative trait loci (QTLs) related to the resistant ability into IR24 under the assistance of molecular makers [25]. So partial genetic base among IR24, 3A26, and 4A37 is identical while 3A26 and 4A37 contained BB resistance QTLs that were mapped on chromosomes 5 and chromosomes 3 separately. Their authentic resistance for BB had been thoroughly verified in Han’s study [25]. All these rice samples were provided by State Key Laboratory Breeding Base for Zhejiang Sustainable Pest and Disease Control, Hangzhou, China. Those rice plants were rigorously reared by block with the size of 2 m × 15 m and a row spacing of 50 cm in the experimental field located at Zhejiang Academy of Agricultural Science, Hangzhou, China. Over the growing stage, sufficient water provision, nutrition supply, and fundamental disease control were guaranteed for decreasing the impact of irrelevant variables.

Two major comparable groups entailing BB inoculation and ultrapure water inoculation were investigated. *Xoo*, a kind of bacterial blight strains, was cultured on the potato dextrose agar medium for proliferation. The optical density (OD_600_) of its bacterial solution which was attenuated by phosphate buffer saline (PBS) solution was supposed to reach 0.8. Artificial leaf tip removing method manufacturing about 3 mm wound tissue with scissors was conducted so that BB could be easily inoculated. The scissors ought to be dipped in the solution of *Xoo* and sterile water before cutting the rice leaf tip. Additional pretreatment procedure was not required before the spectral collection. Inoculation experiments in this study started at the beginning of the rice booting stage on August 22th in 2020, corresponding to the experiment setting for general BB resistance assessment.

### VIS–NIR hyperspectral image acquisition

**Fig. 1:**
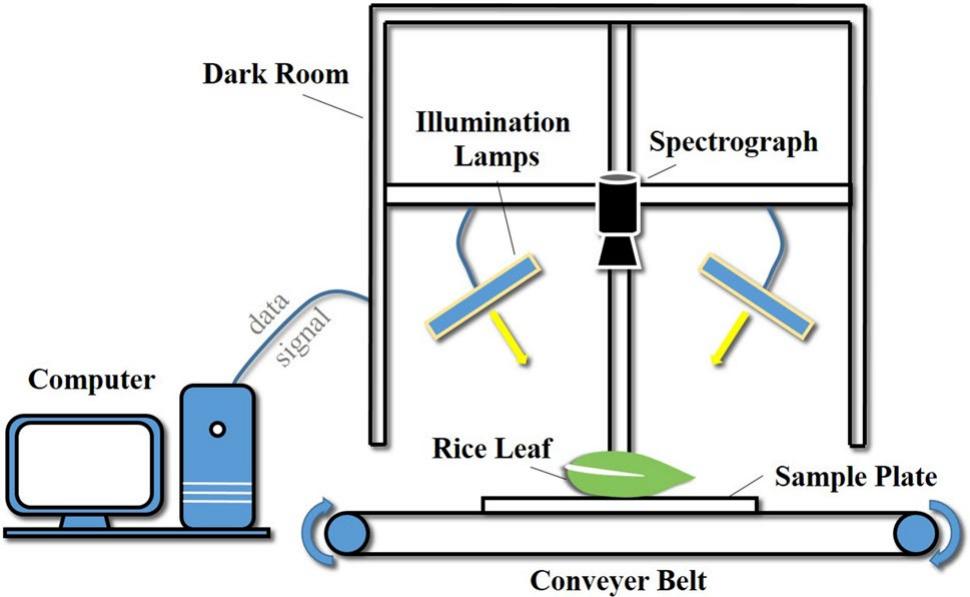
The structure diagram of VIS–NIR hyperspectral imaging system

VIS–NIR hyperspectral images in the range of 413–1016 nm with a total band amount of 473 were obtained by using the laboratory-built hyperspectral imaging system in Zhejiang University, Hangzhou, China (Fig. 1). The whole VIS–NIR hyperspectral image system comprised spectrograph module, illumination module, and mechanical module. Complete rice leaves were necessarily detached from the individual plant with a minimum length of 15 cm for the data acquisition. Leaf samples which were carefully flattened were placed on a plastic plate with a black background for heightening the signal to noise ratio. An electrical motor-driven moving belt took the responsibility to carry the sample plate with a pre-determined moving speed of 2.2 mm/s. At a distance of 29 cm between the sample surface to the spectrograph lens, the pivotal hyperspectral images were taken by an imaging spectrograph (ImSpector V10E; Spectral Imaging Ltd., Oulu, Finland) coupled with a highly sensitive EMCCD camera (Raptor EM285CL, Raptor Photonics limited, Larne, United Kingdom). For the purpose of avoiding image deformation and blur, the exposure time of the camera and the intensity of the illumination module which included two 150 W tungsten halogen lamps (3900 Lightsource, Illumination Technologies Inc., United States) was adjusted to 45 ms and 135. Hyperspectral image correction [Eq. (1)] was carried out after acquiring the bright reference image (the nearly 100% reflectance image of the pure white Teflon board) and the dark reference image (the nearly 0% reflectance image of covered lens) so as to eliminate the nature light influence.

1 $$\begin{array}{*{20}c} {{\text{I}}_{c} = \frac{{I_{r} - I_{d} }}{{I_{b} - I_{d} }} } \\ \end{array}$$

where $I_{r}$ represents the raw spectral image, $I_{d}$ represents the dark reference image, $I_{b}$ is the bright reference image and ${\text{I}}_{c}$ is the corrected hyperspectral image.

Time-series VIS–NIR hyperspectral images of a total of 306 rice leaves from 3 rice varieties were gathered on the 3rd, the 9th, the 15th, the 20th, the 28th, the 33th, the 38th, and the 43rd days after infection. The detailed sample composition was presented in Table 1.

**Table 1:** The detailed sample structure from various experimental groups

Time after infection (day)	IR24		4A37		3A26	
	Sterile water	*Xoo*	Sterile Water	*Xoo*	Sterile water	*Xoo*
3	10	10	10	11	10	10
9	10	10	10	10	10	10
15	5	5	5	5	5	5
20	5	5	5	5	5	5
28	6	5	5	5	5	6
33	5	5	5	5	5	5
38	5	5	5	5	5	5
43	5	5	6	6	6	5

### Spectral feature extraction and disease severity definition

In order to concentrate on the spectral features of regions of interest (ROIs) and reduce computing cost, it was necessary to disregard background noises and average the pixel-wise spectrums of object. Two main procedures involving segmentation and concentration of the rice leaf spectral image and lesion spectral image were ineluctable. In the first place, considering the huge disparity between background and rice leaf, the threshold segmentation method was capable enough to accurately extract leaf spectrum [26]. Pixel-wised spectral information from the background and rice leaf were located by ENVI 5.2. The threshold band and its reflectance value that could separate the objects was determined by means of calculation the L1 distance between their spectrums, according to the equation:

2 $${\text{L}}1\left( {R_{b} ,R_{l} } \right) = \mathop {\max }\limits_{n} \begin{array}{*{20}c} {\left| {R_{b}^{n} - R_{l}^{n} } \right|} \\ \end{array}$$

where $R_{b}$ represented the spectral reflectance value of the background, $R_{l}$ was the spectral reflectance value of rice leaf, and *n* referred to the series number of spectral band. Owing to the pre-experiment, the spectral band at 778.69 nm and the threshold value of 0.11 was eventually chosen to fetch spectral mask of rice leaf. After that, averaged spectrum and pixel-wise area of rice leaf were calculated with the aid of connected component analysis. Secondly, accompanied by the evolvement of irregular BB lesion region, the junction between healthy area and infected area would become more and more mixed generating an obstacle of applying L1 threshold segmentation. And it was substantial to figure out the veritable lesion spectrum to appraise the disease severity. Thus, the specialized expert in BB disease manually labeled leaf lesion regions by utilizing software ENVI 5.2 after which both morphology and spectroscopy information of infected regions and partial healthy regions like pixel area and averaged reflectance value were acquired. Generally speaking, breeding specialist estimates BB disease severity through computing the pixel-wise proportion of lesion area to leaf area, as shown in Table 2, which emphasizes the intrinsical phenotypic difference [27]. Here, for the sake of following feature mining and model interpretability, the ratio of infected area over whole rice leaf area was computed in Eq. (3) and similar disease severity definition was adopted.

3 $$\begin{array}{*{20}c} {P_{l} = \frac{{\sum p_{lesion} }}{{\sum p_{leaf} }} \times 100\% } \\ \end{array}$$

where $P_{l}$ represented lesion proportion over the leaf, $\sum p_{lesion}$ and $\sum p_{leaf}$ severally denoted pixel summation of lesion region and whole leaf region.

**Table 2:** The assessment criteria of *BB* disease severity

Rank	Lesion proportion (%)
0	0
1	0–10
2	10–20
3	20–50
4	50–75
5	75–100

### Data analysis methods

**Fig. 2:**
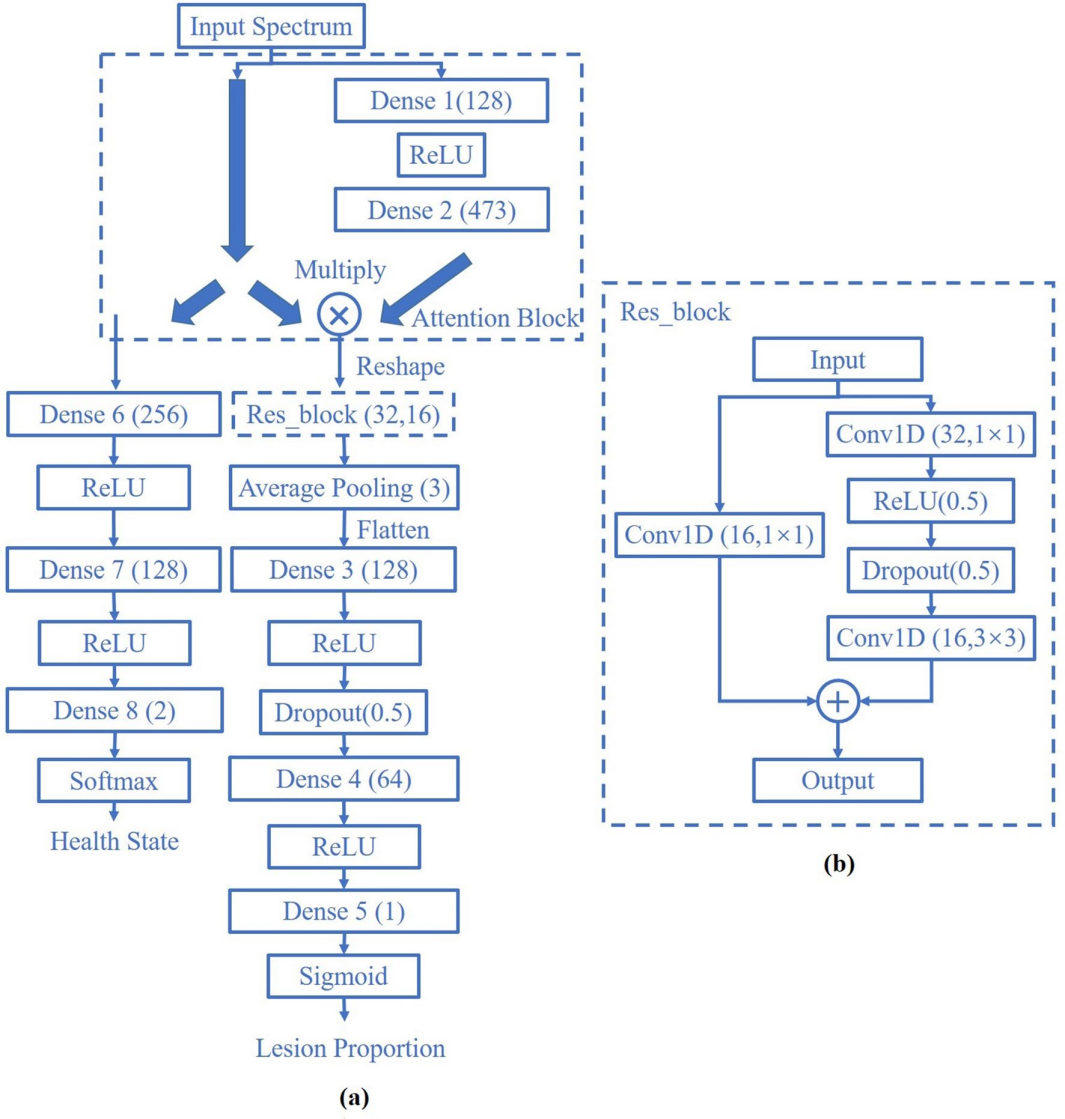
The schematic structure and corresponding parameters of established two-branch LPnet. **a** The complete composition of LPnet; **b** The detailed structure of ResNet block

For fully reveal the specific spectral features which had a distinct association on BB disease severity, a self-built deep learning model, namely two-branch LPnet, was created. The concrete model structure and homographic hyper-parameter were displayed in Fig. 2. Since non-infected experimental groups were involved in the phenotyping assessment, healthy state identification became critical and LPnet was qualified to output hybrid results including the lesion proportion and health state. For the health state classification branch of LPnet, the ground truth label was determined by the experiment setting, for example, water group was labeled as 0 and *Xoo* group was labeled as 1. And ground truth label for this regression branch was settled to be the calculated lesion proportion which was normalized to the range of 0–1.

As for the health state identification branch, the model structure was constructed to be lite and directly receive averaged spectrums from the input without affecting the learning process of the side-branch. Softmax function was performed to transform the output score to logarithmic probability for latter iteration. In the lesion proportion prediction branch, averaged spectrums of whole rice leaf obtained at different inoculation times were all sent into the attention block which was designed to highlight significant features based on simulating visual attention [29]. Parameters of the input neural numbers were highly correlated to the wavelength number of VIS–NIR spectrums. The attention block was defined as:

4 $$\begin{array}{*{20}c} {R_{a} = f\left( {{\varvec{W}}*R_{w} + {\varvec{b}}} \right)} \\ \end{array}$$

5 $$\begin{array}{*{20}c} {R_{ij} = (R_{a} *R_{w} )_{i,j} } \\ \end{array}$$

where $R_{w}$ indicated the whole leaf VIS–NIR spectrum and $R_{a}$ represented the output of attention branch layers. $i,j$ respectively denoted the sample size and spectral band amount, ${\varvec{W}}$ and ${\varvec{b}}$ were the trainable parameters.

Feature extraction had been always an important research field in hyperspectral image analysis [28]. Remarkably, through modeling training progress, the branch layers inside attention block automatically learned and outputted the spectral band’s weight driven by the model task, which was exactly the essence of spectral feature mining. Since not only the locations of spectral wavelengths but also homographic weights were simultaneously confirmed. Convolutional neural network (CNN) with the properties of weight sharing and local sparse perception was adopted to form the backbone for the spectral feature excavation. Furthermore, residual learning blocks which were composed by CNN were able to increase the model depth without increasing burden to optimization [30]. Average pooling layer focused on refining the scale-invariant feature so the quantity of training parameters would not be overly large leading to overfitting. In the end, two fully connected layers combined with activation function, namely Sigmoid, built non-linear regression models to predict the lesion proportion of diverse rice cultivars.

Based on gradient descent theory, deep learning model will automatically seek for the optimal results of the loss function. Cross entropy loss and Mean square error (MSE) were selected to minimize the training loss of classification branch and regression branch, while choosing adaptive moment estimation (Adam) algorithm with a gradient descent step of 0.001 as the optimizer. The tenfold cross validation was performed for the sake of adjusting hyper-parameters. All the trainable weights and bias inside the model were initialized according to uniform distribution for speeding up model convergence. A callback function which was implanted into the training procedure recorded the weights and structure of model at the lowest validation loss and highest validation accuracy during 10,000 training epoch. Training set and testing set were randomly divided at a ratio of 7:3 without intentionally partitioning. During the evaluation phase, typical statistic indicators were chosen, including classification accuracy, the coefficient of determination (R^2^), the root mean square error (RMSE), and the ratio of standard deviation of the prediction set to standard error of prediction (RPD).

### Leaf spectral index selection strategy

**Fig. 3:**
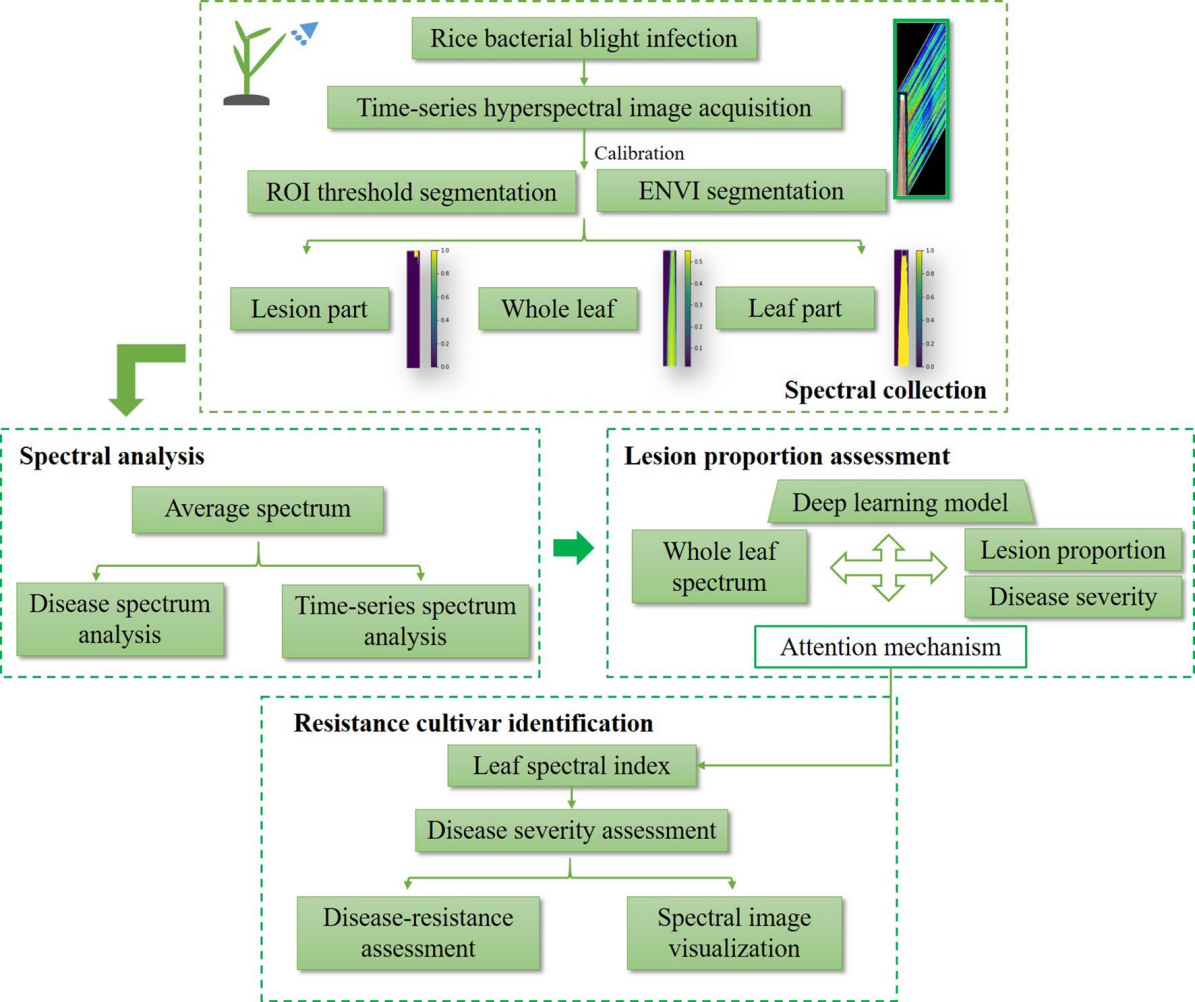
The flow chart of spectrum acquisition and processing

Vegetation indices which are calculated via combining transformation patterns of multiple spectral bands have always been the low-cost and efficient indicator to observe multifarious plant properties including the plant disease [31, 32]. Inspired by the thought of vegetation index, the attention block in LPnet was designed to mine the refined spectral traits for BB lesion proportion and disease severity. As shown in Fig. 2A, directed by the BB lesion proportion prediction assignment, multiplication of the optimized wavelength weights and original spectrums in LPnet was going to make the focal spectral bands stand out. Attention weights that contained positive and negative values were extracted and sorted by size, and spectral bands that possessed top 2 absolute values from both sides and their respective weights were further positioned. Afterward, BB disease severity which had strong connection to lesion proportion could be estimated by calculating relative value of spectral combination towards non-infected rice spectrums, which would be also instrumental for screening the BB resistant cultivar. Several universal vegetation indices (Additional file 1: Table S1) were chosen to compare with the established spectral combination in the value of correlation coefficient (R) with lesion proportion to screen the most competitive, concise, and precise leaf spectral index. The flow chart of data collection and processing in this paper was concluded in Fig. 3.

### Software tools

Involved programming codes for data processing were written and performed on a personal computer whose operating system was Ubuntu 18.04 coupled with Intel(R) Core(TM) i7-8700 K CPU, 3.70 GHz, 16 GB RAM, and GeForce GTX 1080-Ti GPU. Classic ENVI 5.2 was applied to deal with the spectral analysis. The LPnet was created on the foundation of the open source deep learning framework Keras (https://keras.io/) by using Python 3.7.6 (https://www.python.org/). Relevant algorithm code is available on the GitHub address (https://github.com/jinnuozhang/LPnet). Furthermore, all figures were drawn with the help of Origin 9.1 and Microsoft PowerPoint 2016.

## Results and discussion

### Spectral trait

**Fig. 4:**
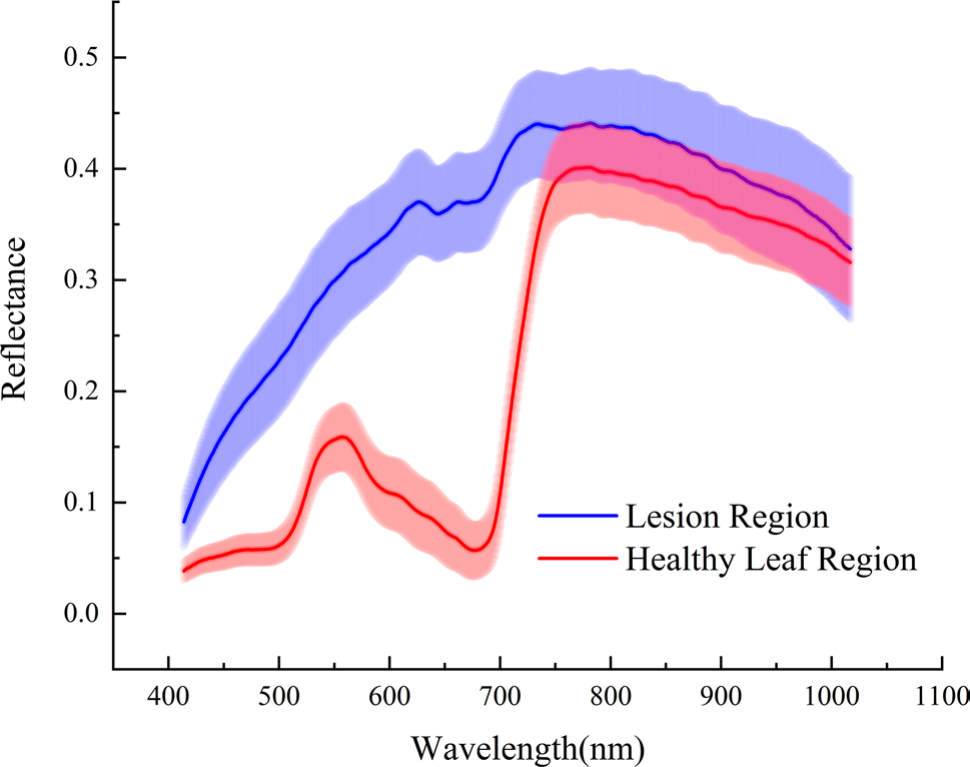
Averaged spectral curves of lesion region and healthy leaf region

Given that the operation of mask extraction, averaged spectrums of partial healthy leaf regions could be fittingly derived through an extra segmentation procedure. Leaving out the variety restriction, spectrums of lesion regions and their partial healthy regions from *Xoo* group under disparate infection time were presented in Fig. 4 along with their standard deviation so as to explore some constitutive spectral differences brought by the BB disease. Apparently, in terms of curve tendency and reflectance values, there were distinct discrepancies between them. What stands out in the figure is that spectrums of lesion regions appeared evidently higher reflectance property in the visible spectral range of 400–750 nm and relative overlapping in the range of 750–1000 nm compared with healthy leaf regions. Prior plant disease related studies that had noted the importance of VIS–NIR spectrums and the similar tendency of lesion spectrum could be found [33–35]. Healthy leaf regions which possessed intact tissue structure tended to accumulate more photosynthetic pigments like xanthophylls, chlorophylls, and carotenoids resulting in a strong absorption in the visible region [36]. While the BB pathogen stressed normal physiological activities, naturally the most sensitive indicators connected to photosynthesis would respond sharply. When it came to the short-wavelength near-infrared spectral range (750–1000 nm), the reflectance values were theoretically related to the multiple frequency vibration of hydrogen-containing groups like O–H [35, 36]. Studies indicated that water content within the plant leaf would be revealed in that range [9, 11]. Hence, it was predictable that withered leaf areas caused by BB pathogen reserved less water content so the reflectance values would become relatively high, which was proved in the exhibition of Fig. 4. Healthy rice leaves possessed comparatively less reflectance in the visible range, which was corresponded with the conclusion in Furlanetto et al. study [12]. According to some previous studies, leaf internal structure damage caused by the bacteria would firstly decrease the spectral reflectance in the near-infrared range [14, 35, 37]. Furthermore, the loss of biomass like water content might eventually cause the uprising reflectance.

From the chart, it could be seen that the lesion spectrum curve had higher statistic dispersion meaning a substantial spectral evolution with the sprawl of BB lesion. Apparently, influenced by the time after infection, there were certain connections between the growing of lesion areas and spectral reflectance variation.

**Fig. 5:**
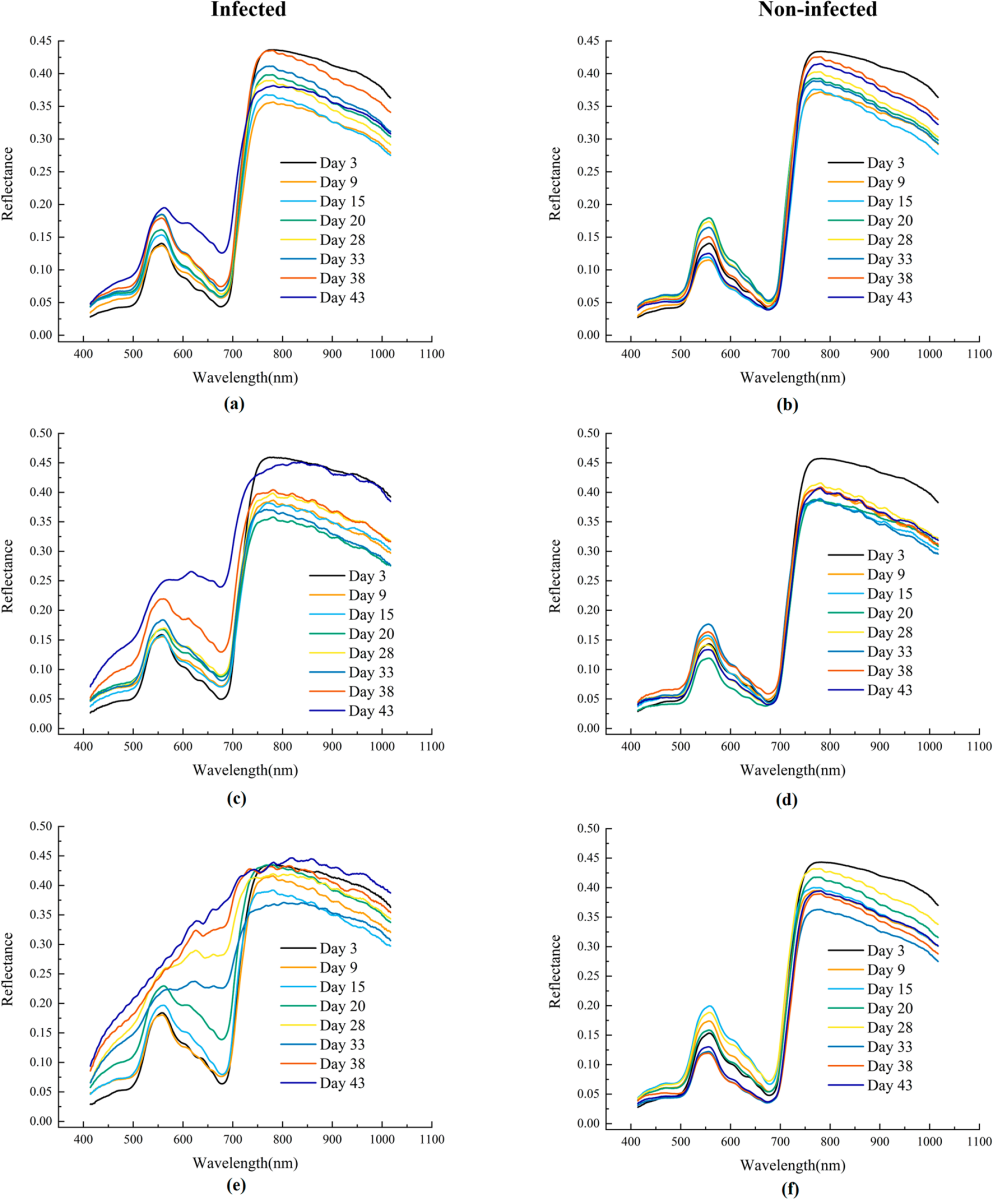
Averaged spectrums of different rice varieties under variant time after infection. **a**, **c**, **e** The infected group, indicating 3A26, 4A37, IR24 respectively; **b**, **d**, **f** The healthy group, indicating 3A26, 4A37, IR24 respectively

Apart from spectrums from separated regions of rice leaves, intact and unified rice leaf spectrums from three varieties were concentrated to figure out the variation pattern under both water treatment and *Xoo* treatment. In Fig. 5 there is a clear diversity of diversity among these three rice varieties. To start with, compared with non-infected spectrums in Fig. 5B, D and F, the increasing of infection time length caused drastic spectral changes in those infected samples, which was consistent with the results in Fig. 4. And the non-infected spectrums appeared equivalent status with spectrums from partial healthy regions of infected spectrums. Based on that phenomenon, it could be challengeable to early detect the BB infection using VIS–NIR spectrums since the majority of regions of the slightly infected leaf were still lively. In Rumpf et al. study, with less than 2% lesion leaf area, the infection identification rate could only reach 65% [38], which implied the difficulty of early detection. What was noteworthy was that in the visible range of spectrums the curve tendency of IR24 bore out its susceptible characteristic (Fig. 5E). With the pathogen infestation prolonging, curves of 3A26 and 4A37 revealed resistant ability holding back the lesion expansion at different levels, which was shown in the form of uptrend speed declining at visible spectral reflectance range. And it was also observed that the spectral reflectance first dropped and then raised in the near-infrared range corresponding to the former analysis. Overall, conspicuous spectral phenotype disparity associated with genotype difference could be found through spectral analysis designating a huge potential of utilizing VIS–NIR spectrum for BB resistance screening.

### Disease severity estimation

**Fig. 6:**
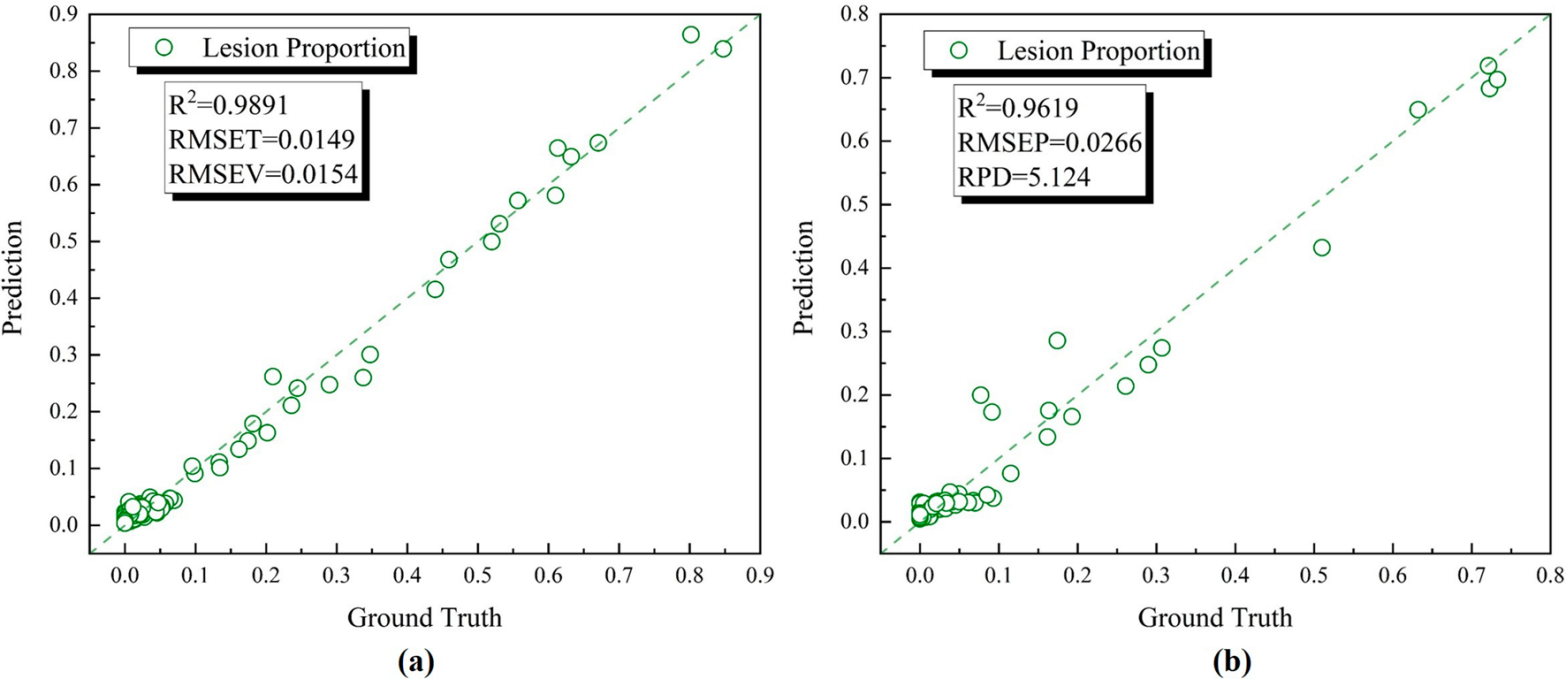
The regression results curves of established LPnet. **a** Training set; **b** Testing set

With the development of withered streak alongside time changing, lesion proportion of these three rice varieties naturally exhibited hierarchical order. Prediction results of both training set and testing set were gathered and made the comparison with the ground truth values. Table 3 provided an overview of the statistical composition of lesion proportion. As presented in Fig. 6, the lesion proportion regression branch outputted promising results that the R^2^ of training set and testing set reached 0.9891 and 0.9619 respectively. What’s more, in consideration of the RMSE value from both training set and testing set, overfitting did not occur which gave a solid proof for stable prediction ability of LPnet. The PRD value which was a reliable standard for the model’s inference performance was calculated to be 5.124 giving solid confidence in the prediction. According to other research, leaf area regression models had been studied and also achieved expected results [39]. The reason for the regression capability of LPnet was likely to be on account of the essential discrepancy of data resource which was composed by lesion spectrum and leaf spectrum and the powerful feature processing ability of deep learning algorithm. The superior performance of our LPnet was further verified by comparison with other leaf area prediction studies [40–42]. Obviously, with the bacterial blight exacerbating, the proportion of disease area increased leading to a dramatically inherent variation inside averaged spectrums. Therefore, under such pivotal theoretical bond, it could reasonably achieve such discernible performance.

**Table 3:** Summary statistics for lesion proportion of rice leaves

Variable	Min	Max	Mean	Std.Dev
Lesion proportion (%)	0	84.76	6.76	15.33

**Fig. 7:**
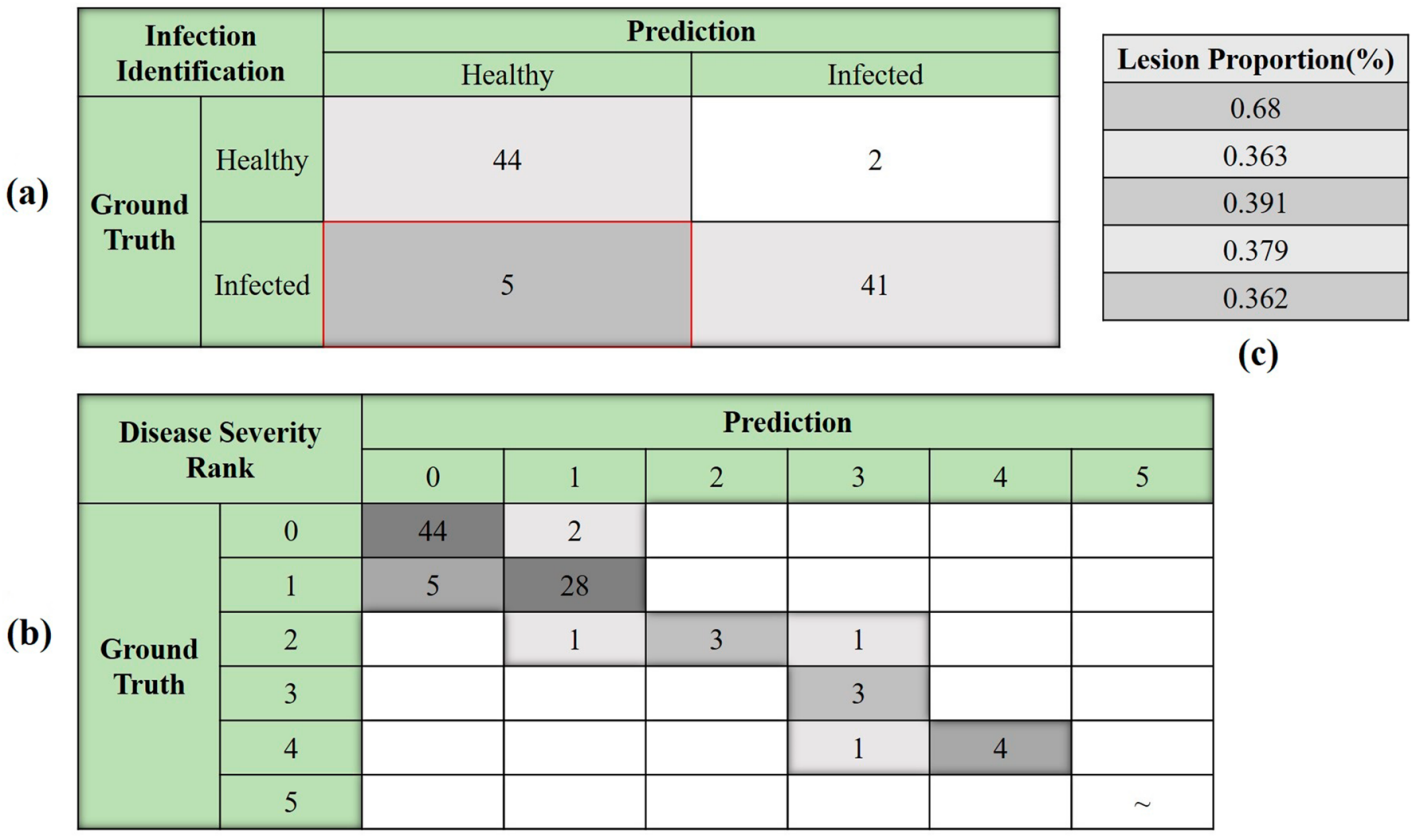
The disease severity estimation results of established LPnet. **a** The classification branch confusion matrix of testing set (accuracy:92.43%); **b** The disease severity estimation confusion matrix of testing set (accuracy:89.13%); **c** The detailed information of misclassification of infected samples

The results of the health state identification from the classification branch of LPnet were summarized in Fig. 7. It is apparent that the correct classification rate of infection achieved 92.43%. By diving into the misclassification profile in Fig. 7C, it could be noticed that minor lesion growth would be hard to make a difference in altering the prediction category toward the infection. These results reflected those of Nguyen et al. who also found that the degree of spectral disparity between healthy samples and infected samples was going to determine the model’s discriminant ability [43]. The estimation accuracy, which was 89.13%, of BB disease severity of all rice leaf samples was set out in Fig. 7B combining lesion proportion regression and infection classification results.

Joint voting mechanism of two separate model branchs was constructed to reduce the estimation errors which were mainly confined to the healthy leaves. For example, without infection identification, due to the flaw of structure of regression branch in LPnet, it was unlikely to produce zero lesion proportion for non-infected samples which was doomed to increasing estimation error. Liang et al. put up with an image-based plant disease estimation network to classify 27 different diseases into 3 severity ranks [44]. Wang el at and Xiao et al. also estimate the leaf disease severity on account of classification results [45, 46]. However, none of these studies had taken the disease severity criterion followed by the breeding expert and time factor into consideration. When it comes to delimiting BB resistant cultivar, lesion proportion is the most generic indicator for breeding expert. There is a congruent relationship between the lesion proportion and resistant peculiarity at a specific time point. The established LPnet was intentionally designed to predict the lesion proportion rather than severity rank for the sake of coping with flexible disease severity standards contrasting to other specified models with restricted output rank amount. Both the interpretability of deep learning model as well as the performance of final evaluation were guaranteed. Taken together, these results provided important insights into exploiting LPnet to mining the hidden features in disease leaf spectrum in view of its excellent performance.

### Leaf spectral index selection

**Fig. 8:**
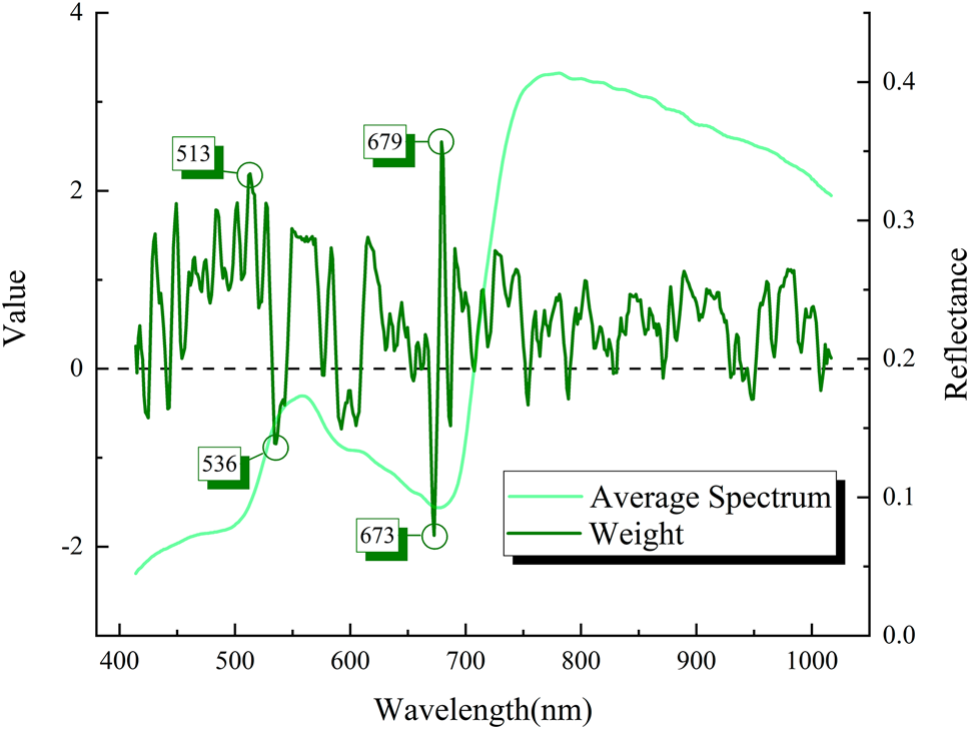
Attention weight curves of LPnet processed by Savizky-Golay filter (window size = 5) and averaged spectral curves of rice leaves

Guided by the attention mechanism and selection strategy, intrinsical spectral features were further refined from the fully trained LPnet to form an effective spectral combination. The neurons of LPnet were activated by the spectrums from training set. As illustrated in Fig. 8, before the channel-wise multiplication operation attention values extracted from the attention block were plotted alongside with leaf averaged spectrum. Conspicuous peaks and valleys of attention curves might elucidate that during the learning progress of LPnet highlighted valuable wavelengths in the form of positive attention and negative attention. Comparison of the finding with those of other studies confirmed that the attention module inside the deep learning structure was able to emphasize informative features in relation to the assigned object [47, 48]. A total of 4 distinct wavelengths and their learned weights were clearly noted, including 513 nm, 536 nm, 673 nm, and 679 nm. The majority of selected bands were located in the visible scope leaving out the near-infrared wavelengths, whose cause might be that undulant near-infrared variation pattern under the BB influence in Figs. 4, 5 had a negative impact on quantifying consistent growth of the lesion. The location of fine-grained spectral wavelengths indicated that leaf pigments related substances had a stronger association with lesion proportion. For instance, wavelengths at around 660 nm usually indicated the existence of chlorophyll a and chlorophyll b [49]. Since spectrums of infected leaves suffered a declining first and uprising next tendency in the range of 780–1000 nm with the infection time increasing, other universal vegetation indices which contained some near-infrared bands inside the formulas tended to inversely change towards the disease infection. The concise and accurate index formula was screened and presented as Eq. (6).

6 $$\begin{array}{*{20}c} {{\text{I}}_{{{\text{LP}}}} = 2.19R_{513} - 0.84R_{536} + 2.55R_{679} - 1.87R_{673} } \\ \end{array}$$

where $R_{513} ,R_{536} ,R_{673} ,R_{679}$ respectively indicted the reflectance values of wavelengths. The corresponding weight meant attention values derived from the LPnet. And ${\text{I}}_{{{\text{LP}}}}$ denoted the calculated leaf spectral index. It was interesting that the equation of the proposed index paid more attention to the variation values since those four bands were located closely.

From the point of view examining the internal structure of deep learning model for better interpretability, attention mechanism takes the responsibility to emphasize valuable features through the attention weighted operation [50]. Attention model based on saliency map outputted numerous attention points which were identical with human sight on testing images [51]. So, it was critical to fully make full use of those extracted attention information. In contrast to earlier studies concerning merely the location of feature wavelengths calculated through original data structure [52, 53], our proposed methods integrated deep learning feature processing ability and spectrum mining.

Vegetation indices like NDVI had been widely used to determine whether the area of interest was infected by disease [54]. By means of measuring the correlation coefficient values between spectral indexes and lesion proportions, comparison between multiple known vegetation indices and proposed I_LP_ was performed in Table 4 by utilizing the whole spectral dataset, and the feasibility and utility of I_LP_ were explicitly validated. It was noticeable that the proposed spectral combination I_LP_ stood out and achieved the highest R value of 0.9660 suggesting a strong relation to lesion proportion. Besides, what was interesting about the data in this table was that without learned attentional weights the relevance between I_LP_ and disease status decreased sharply manifesting the significance of attention mechanism. The development of spectral disease indices based on VIS–NIR spectrums had been investigated by Meng et al. and instead of applying deep learning algorithm, they focused on picking certain features with discriminative power [55]. But there were two individual indices for healthy estimation and infected estimation as well as relatively low qualitative results. Apparently, the proposed I_LP_ was stable and precise enough to compete with other vegetation indices. This kind of laboratory study was going to be transferred into practical practice in the wild environment. In the future, aligned with unmanned aerial vehicle and remote sensing spectrum, the leaf spectral index would make a great difference in high throughput plant disease phenotyping.

**Table 4:** The numerical results of correlation coefficient (R) values between various vegetation indices and lesion proportion

Vegetation index	R value	Vegetation index	R value
I_LP_ with weights	0.9660	MTVI	− 0.7558
I_LP_ without weights	0.4454	PRI	− 0.3020
NDVI	− 0.7671	ZM	− 0.5492
RDVI	− 0.7531	RVSI	− 0.0157
TVI	− 0.7427	HI	− 0.7331
GNDVI	− 0.4833	CI	− 0.6816
OSAVI	− 0.7658	MCARI	− 0.4471
TCARI	− 0.4654	NRI	− 0.7594
SR	− 0.5698	ARI	0.5613
MSR	− 0.6397	SIPI	− 0.7558
TVI	− 0.7494		

### Disease-resistant cultivar identification

**Fig. 9:**
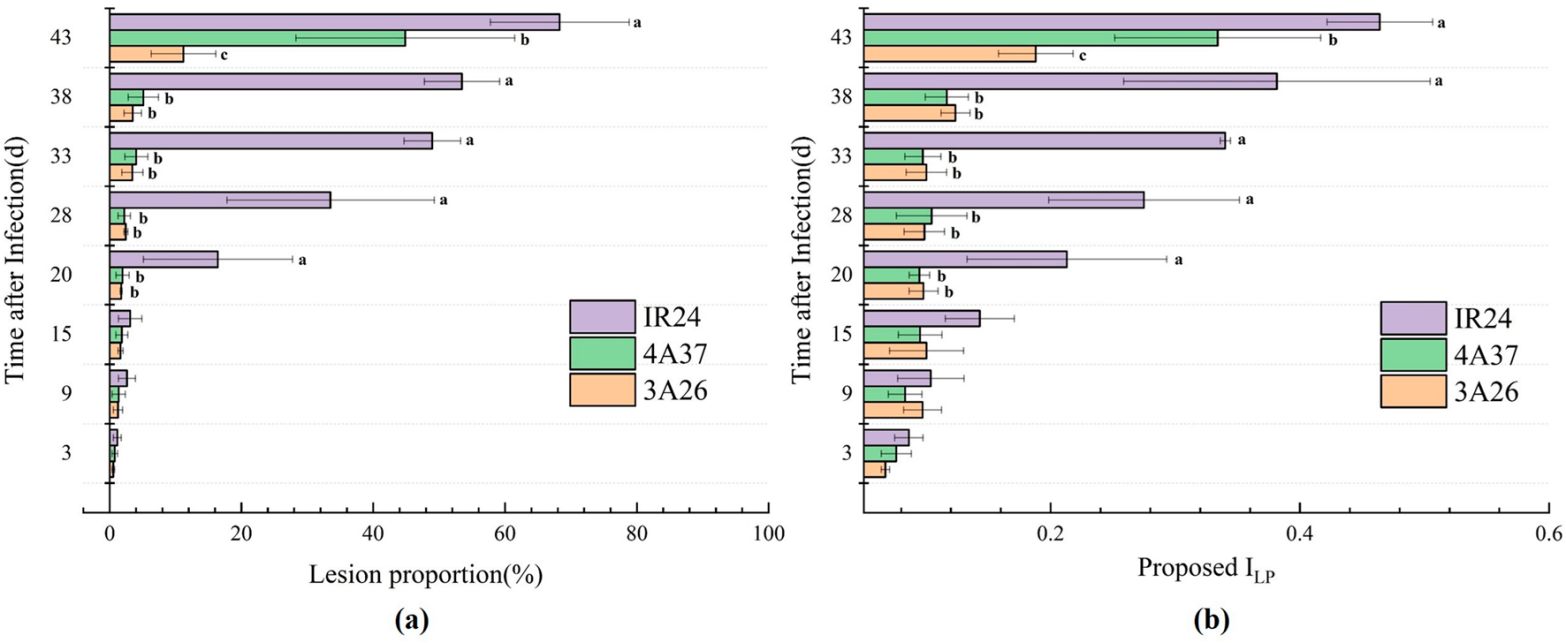
The histograms of lesion area development (**a**) and proposed index values (**b**) at different time after infection and rice variety

In general, taking the lesion proportion as evidence, BB resistant variety was able to be accurately singled out by breeders. In Fig. 9A, both the actual values of lesion proportion and I_LP_ deriving from *Xoo* experimental group were exhibited and compared by rice variety at 8 different times after the inoculation. In the meantime, the results of analysis of variance (ANOVA) which was performed to differentiate whether there were significant differences among those three varieties were labeled by letters (p < 0.05). It was obvious that as the infection period prolonged, the proportion of lesion areas gradually increased, and on the 20th day, there was a significant discrepancy between resistant varieties and susceptible varieties. In addition, there were also evident distinctions between resistant varieties on the 43rd day showing the resistant ability diversity among those cultivars. Taking Fig. 9A as a realistic reference, consistent value variation routine and the intervarietal difference could be observed in Fig. 9B, which meant that the proposed leaf spectral combination could effectively represent the development of BB disease in order to screen resistant varieties and evaluate the resistant ability among those resistant varieties. Although the numerical I_LP_ results were not identical with the ground truth of lesion proportion, the essential features of BB propagation pattern were totally seized.

**Fig. 10:**
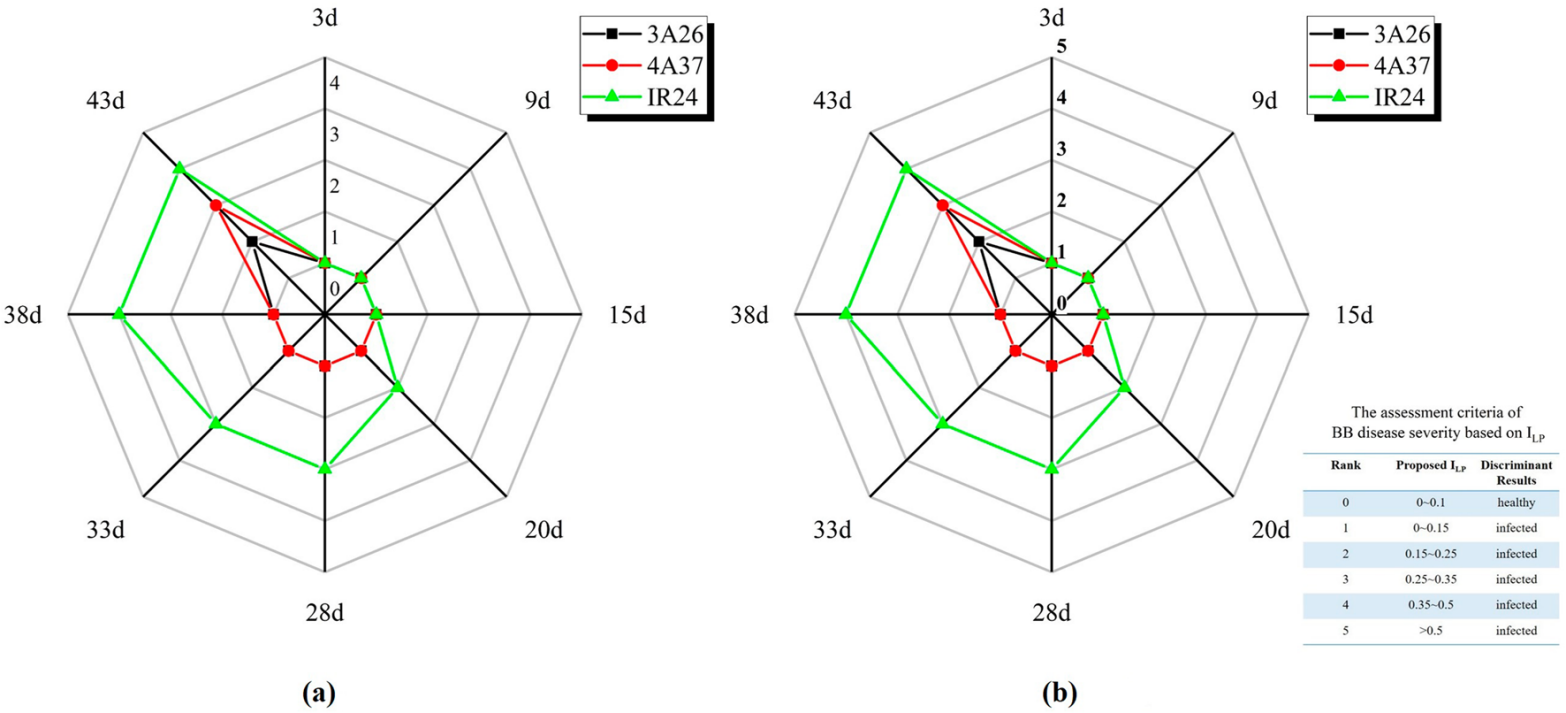
The disease severity estimation radar maps based on the averaged lesion proportions (**a**) and I_LP_ values (**b**) at different time after infection

And when deterministic disease severity was estimated, time-series plant phenotyping would directly point out the right direction of screening the resistant cultivar. Averaged lesion proportions and I_LP_ values from every time point in this experiment were collected and automatically classified into disease severity in Fig. 10. Notably, the determination of rank 0 which represented the healthy samples was fulfilled by fusing the prediction results of the classification branch rather than inventing another spectral index. As the golden reference to determine disease severity, severity rank calculated by lesion proportion appeared similar with ANOVA analysis results. It could be found that the time-series regularity of both lesion proportion and disease severity stayed the same. For instance, on the 20th day, the disease severity ranks of 3A26 and 4A37 stayed at 1 while IR24 reached 2. And at the last experimental date, a totally separate distribution of severity rank among 3 rice varieties could be detected. Despite refined spectral reflectance combination was supposed to be recalculated by the well-designed ranking standard, there was no doubt about its effectiveness and low-budget since it merely took 4 spectral bands.

All in all, with the aid of the proposed I_LP,_ it was accessible to choose an appropriate point-in-time to measure the lesion proportion or assess disease severity, like the 20th day or the 43rd day, discovering the burst and differentiation of BB resistant abilities. In the progress of identifying authentic BB resistant varieties, some hidden information had even been revealed that 4A37 and 3A26 possessed diverse resistant ability. A Study had been done that QTLs in chromosomes 3 (4A37) explained about 26.9% of the resistance variance less than QTLs in chromosomes 5 (3A26) [25], which might result in the diversified phenotyping resistance.

**Fig. 11:**
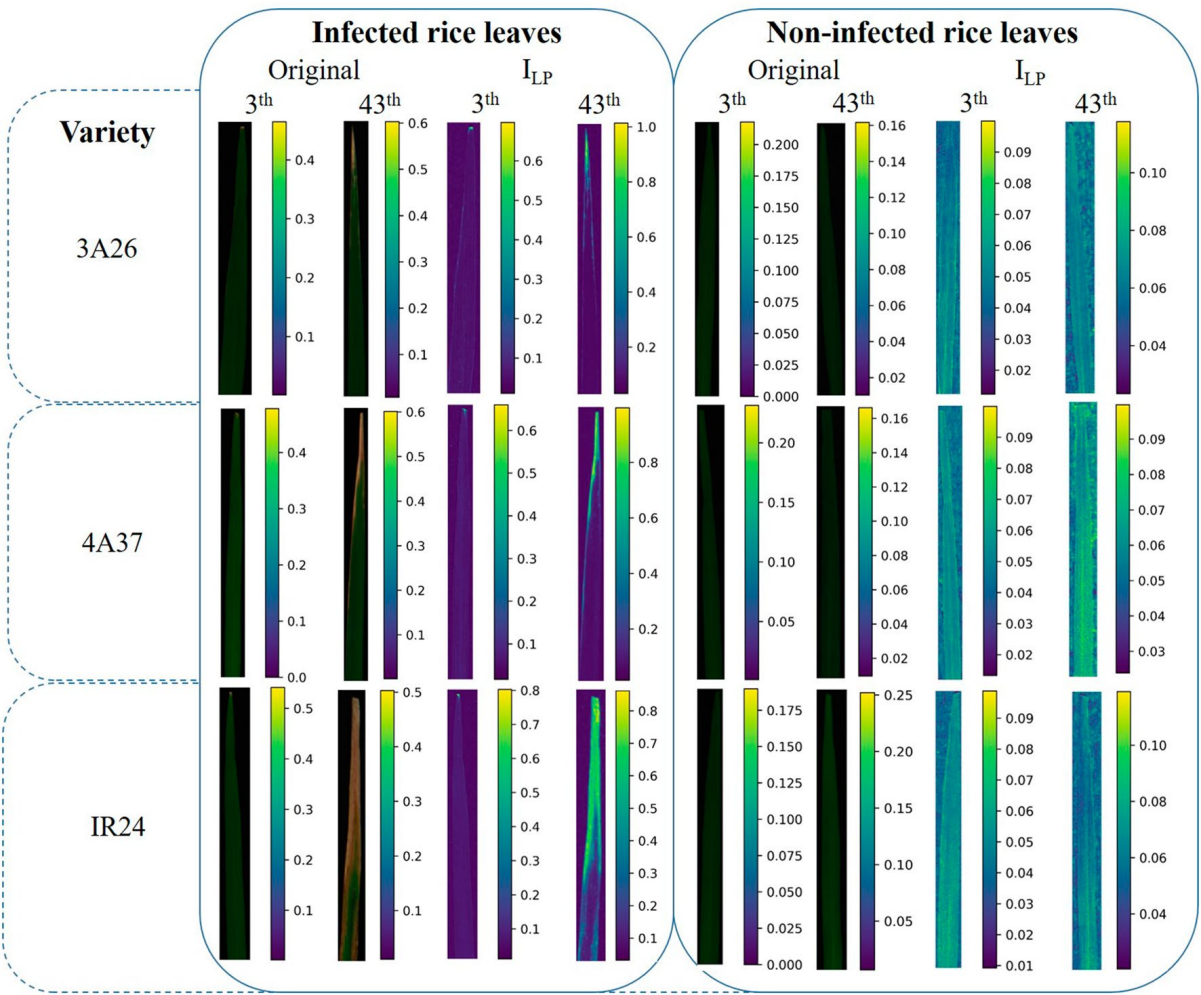
Visualization images of proposed I_LP_ derived from VIS–NIR hyperspectral imaging system

The pseudo-RGB images of rice leaves drawn in three default wavelengths (R channel at 656.03 nm, G channel at 550.71 nm, and B channel at 550.71 nm) and the pseudo-color spectral images calculated based on the leaf spectral index I_LP_ were further visualized in Fig. 11. Compared with the original RGB images, leaf locations with different degrees of infection were highlighted on the I_LP_ visualization images, while the healthy leaves appeared with no obvious highlight areas. And by examining the values on the color bars, it could be found that the larger the lesion proportion value, the larger the calculated value of the proposed index. The diseased leaves would reach 1.0 while the healthy leaves only reached 0.1. The results visualization gave another solid proof for the feasibility of our proposed leaf spectral combination.

## Conclusion

In the current study, time-series averaged VIS–NIR spectrums of rice leaves from different rice varieties, namely 3A26, 4A37, and IR24, at various times after inoculation was collected and analyzed. Spectrums from regions of interest including the whole leaf region, lesion region, and partial healthy region were extracted and calculated to obtain lesion proportions. On account of the high performance and data processing ability of deep learning, a self-built two-branch LPnet was able to identify the health state of input rice leaf spectrums and precisely predict lesion proportions. The classification accuracy and regressive R^2^ of testing set reached 92.43%and 0.9619 respectively. Convincing BB disease severity estimation was achieved by fusing the results of two LPnet branches and the identification rate of testing set achieved 89.13%. Moreover, based on the attention block inside the model, VIS–NIR spectral traits of infected rice leaves were further mining and an innovative leaf spectral index I_LP_ which was proved to be highly related to lesion proportion was proposed, giving a rise to a simple and refined method of assessing disease severity. With the variation of infection time, the effectiveness and feasibility of I_LP_ for identifying the BB resistant variety and assessing the resistant ability was studied and verified that appropriate point-in-time (the 20th day, the 43rd day) to evaluate BB resistant phenotype was determined. Finally, intuitive verification was performed in the form of visualizing the infected and non-infected rice leaves. In conclusion, the proposed LPnet and rice leaf spectral index I_LP_ have great potential in rapidly assisting the disease resistance breeding and precisely excavating the essential phenotype. A further study with more focus on coupling with unmanned aerial vehicle or other portable spectrographs to finish high-throughput rice field phenotyping assessment is advocated.

## Supplementary Information

**Additional file 1: Table S1.** Various vegetation indices calculation formulas.

## Data Availability

Data is available on request to the authors. The deep learning algorithm code is available on the GitHub address (https://github.com/jinnuozhang/LPnet).

## Abbreviations

BB: Bacterial blight
VIS–NIR: Visible/near-infrared
R: Correlation coefficient
R^2^: Coefficient of determination
Faster-RCNN: Faster region convolutional neural network
QTLs: Quantitative trait loci
PBS: Phosphate buffer saline
ROI: Regions of interest
RMSE: Root mean square error
RPD: Ratio of standard deviation of the prediction set to standard error of prediction
ANOVA: Analysis of variance
