# Supplementary material for: Rice bacterial blight resistant cultivar selection based on visible/near-infrared spectrum and deep learning
Source: Plant Methods. 2022 Apr 15;18:49. doi: 10.1186/s13007-022-00882-2 (PMC9013134; doi:10.1186/s13007-022-00882-2)
Supplement: Supplementary file 1 — Additional file 1: Table S1. Various vegetation indices calculation formulas. [file 13007_2022_882_MOESM1_ESM.docx]

**Table S1** Various vegetation indices calculation formulas

| Vegetation Index | Formula | Reference |
| --- | --- | --- |
| Normalized Difference Vegetation Index (NDVI) | $\frac{R_{800}-R_{670}}{R_{800}+R_{670}}$ | [1] |
| Renormalized Difference Vegetation Index (RDVI) | $\frac{R_{800}-R_{670}}{\sqrt{R_{800}+R_{670}}}$ | [2] |
| Enhanced Vegetation Index (TVI) | $\frac{R_{800}-R_{670}}{{1+R}_{800}+{6\times R}_{670}-7\times R_{479}}\times2.5$ | [3] |
| Green Normalized Different Vegetation Index (GNDVI) | $\frac{R_{800}-R_{550}}{R_{800}+R_{550}}$ | [4] |
| Optimized Soil-Adjusted Vegetation Index (OSAVI) | $\frac{R_{800}-R_{670}}{R_{800}+R_{670}+0.16}\times1.16$ | [1] |
| Transformed Chlorophyll Absorption in Reflectance Index (TCARI) | $3\times[{R_{700}-R_{670}-0.2\times(R}_{700}-R_{550})\times\frac{R_{700}}{R_{670}}]$ | [5] |
| Simple Ration (SR) | $\frac{R_{800}}{R_{670}}$ |  |
| Modified Simple Ratio (MSR) | $\frac{\frac{R_{800}}{R_{670}}-1}{\sqrt{\frac{R_{800}}{R_{670}}}-1}$ | [6] |
| Triangular Vegetation Index (TVI) | $0.5\times[{{120\times(R}_{750}-R_{550})-200\times(R}_{670}-R_{550})]$ | [7] |
| Modified Triangular Vegetation Index (MTVI) | $1.2\times[{{1.2\times(R}_{800}-R_{550})-2.5\times(R}_{670}-R_{550})]$ | [8] |
| Photochemical Reflectance Index (PRI) | $\frac{R_{531}-R_{570}}{R_{531}+R_{570}}$ | [9] |
| Red-edge Index (ZM) | $\frac{R_{750}}{R_{710}}$ | [10, 11] |
| Red-edge Vegetation Stress Index (RVSI) | $\frac{R_{714}-R_{752}}{2-R_{733}}$ | [12] |
| Healthy Index (HI) | $\frac{R_{534}-R_{698}}{R_{534}+R_{698}}-\frac{1}{2}\times R_{704}$ | [13] |
| Chlorophyll Index (CI) | $\frac{R_{750}-R_{705}}{R_{750}+R_{705}}$ | [14] |
| Modified Chlorophyll Absorption in Reflectance Index (MCARI) | $\frac{{{[(R}_{700}-R_{670})-0.2\times(R}_{700}-R_{550})]\times R_{700}]}{R_{670}}$ | [15] |
| Nitrogen Reflectance Index (NRI) | $\frac{R_{570}-R_{670}}{R_{570}+R_{670}}$ | [16] |
| Anthocyanin Reflectance Index (ARI) | $\frac{1}{R_{550}}-\frac{1}{R_{700}}$ | [17] |
| Structural Independent Pigment Index (SIPI) | $\frac{R_{800}-R_{445}}{R_{800}+R_{680}}$ | [18] |

**References**

1. Rondeaux G, Steven M and Baret F. Optimization of soil-adjusted vegetation indices. Remote Sens. Environ. 1996;55:95-107.

2. Osborne SL, Schepers JS, Francis DD and Schlemmer MR. Use of spectral radiance to estimate in-season biomass and grain yield in nitrogen- and water-stressed corn. Crop Sci. 2002;42:165-71.

3. Kim Y. Drought and elevation effects on MODIS vegetation indices in northern Arizona ecosystems. Int J Remote Sens. 2013;34:4889-99.

4. Wang FM, Huang JF, Tang YL and Wang XZ. New vegetation index and its application in estimating leaf area index of rice. Chinese Journal of Rice Science. 2007;21:159-66.

5. Yu K, Leufen G, Hunsche M, Noga G, Chen XP and Bareth G. Investigation of Leaf Diseases and Estimation of Chlorophyll Concentration in Seven Barley Varieties Using Fluorescence and Hyperspectral Indices. Remote Sens. 2014;6:64-86.

6. Peddle DR, Brunke SP and Hall FG. A comparison of spectral mixture analysis and ten vegetation indices for estimating boreal forest biophysical information from airborne data. Can J Remote Sens. 2001;27:627-35.

7. Broge NH and Leblanc E. Comparing prediction power and stability of broadband and hyperspectral vegetation indices for estimation of green leaf area index and canopy chlorophyll density. Remote Sens Environ. 2001;76:156-72.

8. Haboudane D, Miller JR, Pattey E, Zarco-Tejada PJ and Strachan IB. Hyperspectral vegetation indices and novel algorithms for predicting green LAI of crop canopies: Modeling and validation in the context of precision agriculture. Remote Sens Environ. 2004;90:337-52.

9. Ye XJ, Sakai K, Sasao A and Asada S. Estimation of citrus yield from canopy spectral features determined by airborne hyperspectral imagery. Int J Remote Sens. 2009;30:4621-42.

10. Zarco-Tejada PJ, Miller JR, Noland TL, Mohammed GH and Sampson PH. Scaling-up and model inversion methods with narrowband optical indices for chlorophyll content estimation in closed forest canopies with hyperspectral data. IEEE T Geosci Remote Sens. 2001;39:1491-1507.

11. Tian YC, Yao X, Yang J, Cao WX, Hannaway DB and Zhu Y. Assessing newly developed and published vegetation indices for estimating rice leaf nitrogen concentration with ground- and space-based hyperspectral reflectance. Field Crops Res. 2011;120:299-310.

12. Perry EM and Davenport JR. Spectral and spatial differences in response of vegetation indices to nitrogen treatments on apple. Comput Electron Agric. 2007;59:56-65.

13. Saviozzi A, Riffaldi R, LeviMinzi R and Panichi A. Properties of soil particle size separates after 40 years of continuous corn. Commun Soil Sci Plan Anal. 1997;28:427-40.

14. Kunz M, Nienartowicz A and Deptula M. The use of satellite remote sensing imagery for detection of secondary forests on post-agricultural soils: A case study of Tuchola Forest, Remote sensing in the 21st century: economic and environmental applications. 2000.

15. Zheng F, Xu B, Xiao P, Zhang X, Manlike A, Jin YX, Li C, Feng X and An S. Estimation of chlorophyll content in mountain steppe using in situ hyperspectral measurements. Spectrosc Lett. 2020;1-12.

16. Riano D, Chuvieco E, Ustin S, Zomer R, Dennison P, Roberts D and Salas J. Assessment of vegetation regeneration after fire through multitemporal analysis of AVIRIS images in the Santa Monica Mountains. Remote Sens Environ. 2002;79:60-71.

17. Barry KM, Stone C and Mohammed CL. Crown-scale evaluation of spectral indices for defoliated and discoloured eucalypts. Int J Remote Sens. 2008;29:47-69.

18. Jiang JB, Steven MD, He RY and Cai QK. Comparison and Analysis of Hyperspectral Remote Sensing Identifiable Models for Different Vegetation under Waterlogging Stress. Spectrosc Spect Anal. 2013:33:3112-6.
